# Contraception for married adolescents (15–19 years) in India: insights from the National Family Health Survey-4 (NFHS-4)

**DOI:** 10.1186/s12978-021-01310-9

**Published:** 2021-12-20

**Authors:** Ijyaa Singh, Ankita Shukla, Jissa Vinoda Thulaseedharan, Gurpreet Singh

**Affiliations:** 1 Achutha Menon Centre for Health Science Studies (AMCHSS), Sree Chitra Tirunal Institute for Medical Sciences and Technology, Trivandrum, Kerala India; 2 Population Council, New Delhi, India

**Keywords:** Adolescent, Family planning, Contraception, Pregnancy, Health care workers, NFHS

## Abstract

**Purpose:**

Despite the fact that marriage below the age of 18 years is illegal in India, a considerable number of females get married and start childbearing during their adolescent years. There is low prevalence of contraceptive methods and high unmet need for family planning (FP). Realizing this, new government programs have been launched to increase the uptake of sexual and reproductive health services among adolescents. However, evidence specific to this age group remains scarce.

**Aim and objectives:**

The present study was conducted to assess the prevalence of modern contraceptives among married adolescents, and to determine its association with sociodemographic variables, health worker outreach, and media exposure to FP messages in India.

**Methods:**

Data for this analysis was drawn from the fourth round of the National Family Health Survey (NFHS-4) conducted in India during 2015–16. The sample size is restricted to 13,232 currently married adolescent girls aged 15–19 years, who were not pregnant at the time of the survey. Bivariate and multivariate analysis were conducted to assess the levels of contraceptive use and its predictors among married adolescents.

**Results:**

The use of modern contraceptives among married adolescents increased from 4 to 10% between 1992–93 and 2015–16. The uptake of modern contraceptives was found to be low among the uneducated, those residing in rural areas, among backward classes, those practising Hindu religion, women in the poorest wealth quintile, women without children, and those with no exposure to FP messages via media or health care workers. Among those who met health care workers and discussed FP issues with them, 34.11% were using modern contraceptives as compared to 11.53% of those who did not have discussions with health care workers.

**Conclusions:**

The evidence suggests that contact with health care workers significantly influences the use of modern contraceptives. Further focus on increasing contact between married adolescents’ and health care workers, and improving the quality of counselling will protect adolescents from early marriage and pregnancy.

## Plain language summary

Complications related to early pregnancies and childbirth are a leading cause of death among adolescent girls. Still, a considerable number of young girls in India get married and start childbearing in adolescence. Data shows that 9% of the girls aged 15–19 years in rural areas and 5% in urban areas have already begun childbearing. The World Health Organisation (WHO) recommends access to high-quality sexual and reproductive health information and services as a fundamental right of adolescents. In recent years, the Indian government has also taken several initiatives to cater to the needs of adolescents.

The present study focuses on the levels of contraceptive use and the influence of health care workers and media outreach on the uptake of modern contraceptives among married adolescents in India.

Findings from the present study suggest that though there has been an increase in the use of modern contraceptives among married adolescents over time, overall the level of contraceptive use is very low. There is visible variation in the use of modern contraceptives depending on the educational status, residence, religion, caste, and household wealth of adolescent girls. Contact of married adolescents with health care workers positively influences the uptake of modern contraceptives. However, the proportion of married adolescents who had contact with health care workers is very low.

## Introduction

India constitutes 20% of the world's 1.2 billion adolescents; of these, at least 1.5 million girls get married each year before they turn 18 years old [1]. Marriage during adolescent years exposes girls to a higher risk of pregnancy-related complications, resulting in higher maternal mortality [2]. Data suggests that 9% of the adolescent girls in rural areas and 5% in urban areas have already begun childbearing in India [1]. High level of unmet need for FP is seen among the age groups of 15–19 years and 20–24 years (27% and 22%, respectively) as compared to the national average (13%) [3].

Similar patterns of higher unmet need for FP and the burden of unplanned pregnancies among married adolescents as compared to older women are seen in other low- and middle-income countries [4]. Across the globe, universal access to reproductive and sexual health services, including FP, is increasingly recognised as a public health priority for improving the health outcomes of adolescents [5]. Despite introducing multiple initiatives for adolescent reproductive and sexual health, the progress in improving health outcomes among adolescents has been slow and inconsistent, and calls for more focussed attention towards the contraceptive needs of adolescents [6].

Along with access, the issue of quality of care in FP services has become a central concern for the international FP community. Better quality of FP services is considered key to meeting the demand for FP and maximizing client satisfaction [7, 8]. Though the quality of FP services is associated with a higher uptake of contraceptives [9], small scale studies from India reveal that quality of FP services is low in the country [10, 11].

Often it is found that young girls either have no knowledge about FP services or have misconceptions about contraceptives that leads to low use of modern contraceptives among them [12, 13]. For the same, generating awareness and demand among young and low parity women through health care worker outreach and media campaigns has remained an important component of the government’s ongoing FP program.

Exposure to media sources such as television and newspapers is associated with higher likelihood of using modern contraceptive methods among adolescents [14]. Research from various parts of the world shows that the role of frontline health care workers is crucial in increasing uptake of FP services by the community and for higher met need of FP [15–17]. Studies from India too have shown that health care workers play an important role in increasing acceptance of FP and RMNCH service acceptance. However, their interaction with FP clients has been limited, particularly in rural areas [18, 19].

In recent years, Government of India has taken several initiatives to cater to the needs of adolescents. The Adolescent Reproductive and Sexual Health (ARSH) strategy was initiated to improve awareness and access to reproductive health services among adolescent youth [20]. At present, the government’s FP program focuses more on young newly-married couples receiving counselling from community health workers to ensure the delay of first birth after marriage and spacing between the first and second births. In 2016, Mission Parivar Vikas (MPV) was launched to increase the access to contraceptives and FP services in 146 high fertility districts. Under this, promotional schemes such as Nayi Pehel Kit (an FP kit distributed through ASHAs to newly married couples) and Saas Bahu Sammelans (to facilitate and encourage communication between young married women and their mothers-in-laws, to freely discuss matters related to FP and reproductive health) have been initiated by the Ministry of Health and Family Welfare [21].

To facilitate any FP program and government strategies, accurate information is required for measuring progress and differentials in the use of modern contraceptives. Though there are a plethora of studies on examining determinants of contraception use among married women in India, very few of these provide age specific details on women aged 15–19 years. As, even now, many girls in the country start their reproductive journey below the age of 18 and have relatively high unmet need for contraceptives, it is imperative for policy makers and program managers to understand their need for FP services and factors influencing their needs.

The present study was undertaken to assess the prevalence of modern contraceptive use among married adolescent girls in India, and determine its association with socio-demographic variables such as education, parity, area of residence, and household wealth. Also, the present study attempted to examine the role of health care workers and FP media outreach in the uptake of modern contraceptives among married adolescent girls in the country.

## Methods

### Data source

Data for this study was drawn from the Indian nationally representative household survey, the National Family Health Survey (NFHS). Four rounds of NFHS were carried out across all the states and union territories of India in 1992–93, 1998–99, 2005–06, and 2015–16, respectively. The NFHS aims to provide information on reproductive health care services, fertility, childhood mortality, knowledge of HIV and contraceptives, nutritional status of the mother and the new-born, among other parameters. The fourth round of NFHS (2015–16) covered 723,875 eligible women aged 15–49 years in 572,000 households. Interviews were conducted for 699,686 women, with a response rate of 97%. Among them, 16,864 currently married adolescent girls aged 15–19 years were interviewed in the 4th round of NFHS. The present analysis was restricted to 13,232 currently married adolescents aged 15–19 years who were not pregnant at the time of the survey.

The Demographic and Health Survey (DHS) in India is known as NFHS. Procedures and questionnaires for standard DHS surveys have been reviewed and approved by the ICF Institutional Review Board (IRB). Additionally, country-specific DHS survey protocols are reviewed by the ICF IRB and typically by an IRB in the host country. ICF IRB ensures that the survey complies with the U.S. Department of Health and Human Services regulations for the protection of human subjects (45 CFR 46), while the host country IRB ensures that the survey complies with the laws and norms of the nation.

### Outcome variables

The use of modern contraceptive methods (mCPR) was considered as the key outcome variable. Currently married adolescents using any of the modern contraceptive methods (condom, pill, IUD, injectable, female/male sterilization, or other modern methods) at the time of the survey were coded “1”, otherwise “0”.

### Independent variables

Socio-demographic variables in the present study included education (no education, 1–5 years, 5–10 years, and more than 10 years); place of residence (urban, rural); caste (scheduled caste/scheduled tribe [SC/ST]), other backward castes [OBC], and other); religion (Hindu, Muslim, and other); household wealth index (a composite score given in the NFHS based on household assets categorized into five categories: poorest, poorer, middle, richer and richest), have a child (don’t have children, have children). Further, health care workers’ outreach to married adolescents for providing FP information was considered as one of the key independent variables in the study. This study captured health worker's outreach for FP using the following two variables: (1) health workers contacted adolescents in the past three months, and (2) health workers contacted adolescents in the past three months and discussed FP. Here the contact between adolescents and health care workers took place in-person at the adolescent’s home or in the health centres. Lastly, media exposure to FP messages was considered as present if the adolescent had heard FP messages on at least one of the media platforms such as radio, TV, newspapers, or magazines.

### Data analysis

Univariate and bivariate analysis was conducted to examine the levels of contraceptive use and unmet need among married adolescents over time. Chi-square test was used to understand the association between modern contraceptive usage and various sociodemographic characteristics of married adolescents. Multivariate logistic regression was conducted to understand the relationship between background characteristics, media exposure to FP messages, and health care worker's outreach on the usage of modern contraceptives among adolescents. Results from the regression analysis were presented in terms of adjusted odds ratio (AOR), with a 95% confidence interval (CI) and significance level. Interpretation of the adjusted odds ratio can be as, if AOR > 1, then there are higher odds of using contraceptives. The analysis performed was carried out using the analytical software STATA 14.1.

## Results

**Fig. 1:**
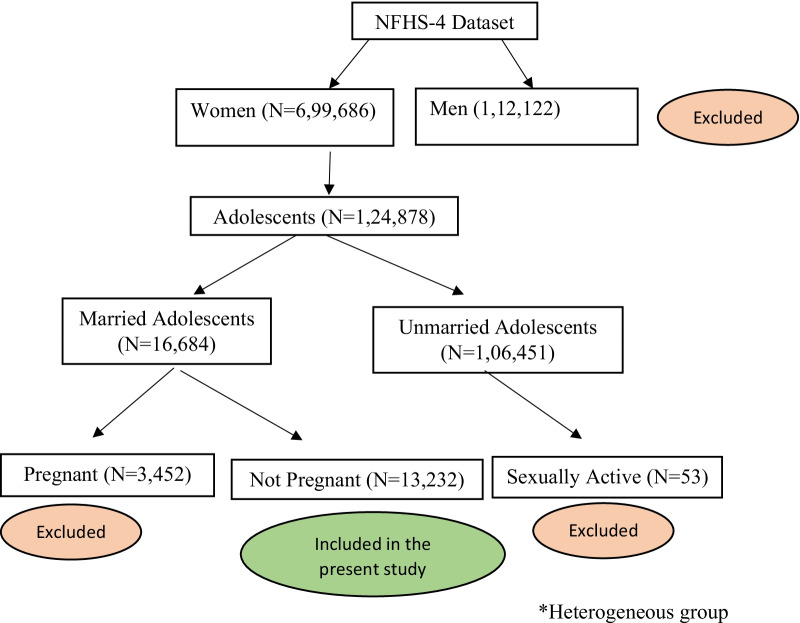
Schematic representation of sample selection from NFHS-4

A schematic representation of the NFHS datasets and included records for the present study are shown in Fig. 1. A total of 13, 232 married adolescents were included in the study.

### Current use of family planning among married women across all age groups in India

**Fig. 2:**
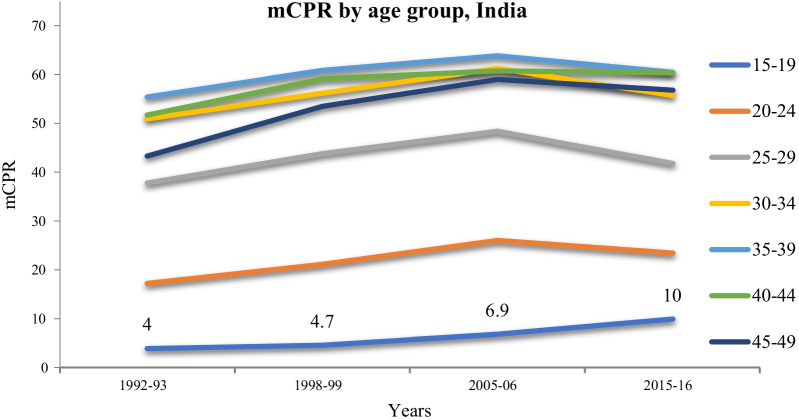
Age wise distribution of Modern contraceptive use (mCPR) among married adolescents, (1992–2016), India

The current use of modern contraceptives among married adolescents (15–19 years) increased from 4% in 1992–93 to 10% in 2015–16 (Fig. 2). Though girls in this age group have the most need for contraceptives, the current use of contraceptives was considerably lower among married adolescents compared to married women in older age groups.

Between NFHS-1 and NFHS-4, the use of condoms and pills increased considerably, whereas it has been stagnant for IUD (Table 1). The use of traditional methods among married adolescents increased between NFHS-1 to NFHS-3 (from 4 to 6%) but declined to 5% in NFHS-4. However, in NFHS-4 majority of adolescents used a modern method (10%), but 5% still relied on traditional (and presumably less effective) methods. Though the unmet need for FP declined in this group, almost a quarter of married adolescents had an unmet need for contraceptives as per NFHS-4.

**Table 1:** Contraceptive use by type of method among married adolescents aged 15–19 years, (NFHS 1–NFHS 4), India

	NFHS 1 (%)	NFHS 2 (%)	NFHS 3 (%)	NFHS 4 (%)
Current contraceptive use any method (CPR)	7.1	8	13	14.9
Current contraceptive use modern method (mCPR)	4.0	4.7	6.9	10.0
Female sterilization	1.3	1.5	1.1	0.9
Male sterilization	0.0	0.0	0.0	0.0
IUD	0.6	0.5	0.3	0.5
Pill	0.8	1.3	2.2	3.9
Condom	1.2	1.4	3.3	4.4
Other	0.1	0.0	0.0	0.3
Traditional methods	3.9	3.3	6.0	4.9
Unmet need of FP*		27.1	27.1	25.0

*Not available

### Health care worker contact and media exposure on FP messages among married adolescents girls in India

**Fig. 3:**
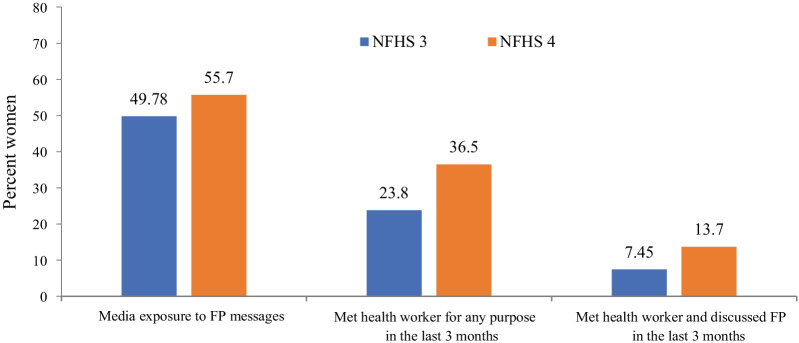
Health care worker contact and media exposure on FP messages (NFHS-3 & NFHS-4)

Figure 3 shows an increase in trend of media exposure (49.78 to 55.7%) and health care workers outreach (7.45 to 13.7%) for FP messages from NFHS-3 to NFHS-4. Just over half of the married adolescents (55.7%) had heard FP messages on television, radio, or newspapers/magazines. Thirty-seven percent of married adolescents were contacted by the health care workers in the past three months; however, discussions on FP during this contact took place only in 13.7% of the cases.

### Use of modern contraceptives by background characteristics of married adolescents as well as the effect of health care workers’ outreach

The percentage of modern contraceptive use was found to be higher among educated adolescents as compared to those without formal education (12.21% vs. 8.02%, respectively, p < 0.05) (Table 2). Also, the modern contraceptive uptake among adolescent girls in the poorer wealth quintile was lower as compared to those in the richest quintile (9.64% vs. 15.87%, respectively). Adolescent girls who belonged to the SC/ST group had less usage of modern contraceptives as compared to other castes (12.0% vs. 19.69%). Modern contraceptive use was 11.34% among adolescent girls belonging to the Hindu religion, which was low as compared to contraceptive uptake in the Muslim religion (17.46%). Adolescent girls from a religion other than Hindu and Muslim have higher usage of modern contraceptives (20.15%). Adolescent girls who already had children had a higher uptake of modern contraceptives as compared to those without any children (20.52% vs. 7.72%). Among those who were exposed to media FP messages, 13.39% were using modern contraceptives. The use of modern contraceptives was 34.11% among married adolescents who met and discussed FP with health care workers, while 11.53% among those who did not have contact with health care workers.

**Table 2:** Percentage of married adolescents aged 15–19 years using modern contraceptive methods by selected background characteristics and program outreach, NFHS-4, India

Characteristics	N	mCPR	P-value*
*Education*			
Uneducated	2322	8.02	
1–5 years	1664	13.02	
5–10 years	7053	14.06	
10 + years	2193	12.21	< 0.001
*Place of residence*			
Rural	11,036	11.99	
Urban	2196	15.36	< 0.001
Caste*			
SC/ST	5241	12.01	
OBC	5396	9.1	
Others	2009	19.69	
*Religion*			
Hindu	13,055	11.34	
Muslim	2582	17.46	
Others	1227	20.15	< 0.001
*Household wealth*			
Poorer	3752	9.64	
Poorest	3908	14.14	
Middle	2876	12.92	
Richer	1846	12.97	
Richest	850	15.87	< 0.001
*Have at least one child*			
No	8018	7.72	
Yes	5214	20.52	< 0.001
*Media exposure to FP messages*			
No	5950	11.67	
Yes	7282	13.39	< 0.001
*Health care worker contacted in the past 3 months*			
No	9,424	9.78	
Yes	3,808	19.41	< 0.001
*Health care worker contacted in past 3 months and discussed FP*			
No	12,668	11.53	
Yes	564	34.11	< 0.001

*Indicates p-values for chi-square test, N = unweighted sample in each category

The results from the logistic regression model are represented in Table 3. The odds of modern contraceptive use among adolescent girls with > 10 years of education was 1.55 times (p < 0.05; 95% CI: 1.09–2.19) higher than uneducated girls. The adjusted odds ratio of using modern contraceptives was higher among adolescent girls belonging to other religion (AOR: 1.56, p < 0.05; 95% CI: 1.06–2.28) and those from the richest wealth quintile (AOR: 1.82, p < 0.05; 95% CI: 1.21–2.73). Furthermore, adolescent girls with at least one child had higher odds of using modern contraceptives (AOR: 2.72, p < 0.05; 95% CI: 2.22–3.33) as compared to those who did not have a child yet. The adjusted odds ratio of using contraceptives was 3.01 (p < 0.05; 95% CI: 2.14–4.22) among adolescent girls who had contact with health care workers and discussed FP as compared to those who did not.

**Table 3:** Association between selected background characteristics and use of modern contraceptives among married adolescents aged 15–19 years, NFHS-4, India

Characteristics	Adjusted OR	CI
*Education*		
Uneducated	1	
1–5 years	1.59**	(1.13,2.24)
5–10 years	1.59**	(1.20,2.12)
10 + years	1.55**	(1.09,2.19)
*Place of residence*		
Rural	1	
Urban	1.24*	(0.97,1.58)
Caste*		
SC/ST	1	
OBC	0.74**	(0.61,0.90)
Others	1.57**	(1.22,2.01)
*Religion*		
Hindu	1	
Muslim	1.11	(0.85,1.46)
Others	1.56**	(1.06,2.28)
*Household wealth*		
Poorer	1	
Poorest	1.49**	(1.08,1.79)
Middle	1.27*	(0.96,1.68)
Richer	1.29	(0.93,1.79)
Richest	1.82**	(1.21,2.73)
*Have at least one child*		
No	1	
Yes	2.72**	(2.22,3.33)
*Media exposure to FP messages*		
No	1	
Yes	1.07	(0.89,1.29)
*Health care worker contacted in the past 3 months*		
No	1	
Yes	1.07	(0.86,1.33)
*Health care worker contacted in past 3 months and discussed FP*		
No	1	
Yes	3.01**	(2.14,4.22)

*Indicates p-value < 0.010 and **indicates p-value < 0.005

## Discussion

A comparison of NFHS data from four rounds of the survey conducted in 1992–93, 1998–99, 2005–06, and 2015–16 enables us to develop longitudinal insights regarding trends in modern contraceptive use among married adolescents aged 15–19 years. According to the present study, the contraceptive usage among married adolescent girls has remained consistently low, as compared to higher age groups. Though there has been an increase in the use of modern contraceptive methods from 1992 to 93 (4.0%) to 2015–16 (10%) among married adolescents, the progress in this age group has been slow, as compared to older age groups (20–49 years). It is estimated that 9.1% of adolescent girls (aged 15–19 years) used modern methods of contraception globally in 2019 [22]. The use of modern contraception was higher in Northern America and Europe and lower in Western Asia and Northern Africa.

The present study also reveals that married adolescents have a high dependence on traditional methods. Among those who reported using any method, 33% relied on traditional methods. This is higher than the global average where majority of adolescent users (89%) report using a modern method [22]. The most preferred modern method among adolescents was condoms, followed by pills. The use of long-term modern methods such as IUD is almost negligible and has shown no progress over the years. It is in line with the finding that in low- and middle-income countries, almost 89% of modern methods users in the adolescent age group use short-term acting methods [20]. This may be attributed to the fact that among adolescent women, there is lack of adequate knowledge on FP and wide spread misconceptions regarding the side effects that modern contraceptives may have on their ability to bear children in the future [23, 24].

In cultural settings like India, it is often found that there is societal pressure to conceive the first child soon after marriage to prove fertility [25]. Proving fertility immediately after marriage is seen as an important factor in stabilising marital relationships and being respected by the in-laws. Despite efforts by the government to focus on sexual and reproductive health care needs of adolescents, young married women, and low parity women, suggestions by health care providers are often influenced by the existing social norms [10]. A recent study from India found that health care providers were hesitant to provide/suggest contraceptive methods to young and zero parity women [10]. These providers were concerned about the side effects associated with methods that can hamper the fertility of women. Moreover, young married women are known to have restricted autonomy and very low exposure to social networks [23, 26]. Married adolescents lack negotiation abilities and consequently the decisions about their life are largely made by either their husbands or mothers-in-law [23]. Due to early marriage, education of these adolescents is hampered, thus restricting their ability to be financially independent and reducing their exposure to information about their reproductive health.

Results suggest that being uneducated, poor, residing in rural areas, and having no children decreased the likelihood of using modern contraceptives among adolescents. This was found to be similar to previous studies that have identified the role of socio-demographic characteristics as facilitators and barriers for uptake of modern contraceptive use among adolescents [27–29]. Poor and less educated adolescent girls have been found to have limited autonomy, knowledge, and awareness about contraceptive use [30]. Women who get married late are able to complete their education and have better knowledge about contraceptive usage [31].

The present study found a positive association between contact with health care workers and use of modern contraceptive methods. Those who had met a health care worker and discussed FP with them had higher odds of using modern contraceptives. To improve adolescents’ awareness and access to contraception, Government of India has launched several initiatives in recent times, such as the provision of village health nutrition days, designated ARSH counsellors in public health facilities, and distribution of Nayi Pehel Kits to increase the contact between health workers and adolescents [32]. However, present results reveal that only 36% of married adolescents reported having discussions about FP.

Limited access to sex education for girls in school as well as at home, limited freedom to access contraceptive clinics [33], and negative behaviour of the providers [10] are known to be prominent barriers to use of contraceptives among married adolescents. Thus, it is recommended to widen the routes of information on sexual and reproductive health to empower adolescent girls in making informed decisions for themselves.

A good example is the PRACHAR initiative implemented in Bihar, an Indian state with high fertility and high prevalence of early marriages. The PRACHAR initiative was aimed to delay the age at first birth by delaying the age at marriage and increasing voluntary contraceptive use among young nulliparous married women. Under the program, adolescents were trained in communication skills to negotiate with partners and parents in order to achieve their reproductive goals. Messages were disseminated through street theatre performances and wall paintings; and formal and informal rural health service providers were trained on reproductive health issues and contraception [34]. Learnings from the PRACHAR Project in Bihar indicates the importance of comprehensive programming with gender-synchronized interventions tailored to specific life stages [35]. Decisions about marriage and fertility are not taken in isolation and are equally important for both boys and girls. Hence, community level interventions focused on both boys and girls can bring behaviour change regarding early marriage and FP.

Present findings suggest that though there are multiple initiatives and programmes for adolescents in the country, there is a need to relook social and behaviour change communication strategies carried out by the health care workers. It is a well-established fact that the quality of FP discussions significantly impacts acceptance rates of contraceptives in the target population [36]. Unfortunately, evidence from India suggests that quality of FP care and client-provider interaction is not satisfactory [10]. Such learnings indicate that to ensure the success of ongoing programs, there is a need to emphasize more on the training given to FP providers. An important addition to the training of service providers should be strengthening their skills in counselling adolescent girls on how to prevail over existing social norms of the community that impede their decision-making power, especially in the context of their sexual and reproductive health.

NFHS-4 is a large scale multi-round survey, that collects data with a high response rate in urban and rural areas across all 36 Indian states and union territories utilising standard protocols and quality control processes. Use of the NFHS data in the present study provides robust national level estimates. However, the present study had certain limitations. There are additional factors which impact contraceptive use such as family dynamics, social norms, and quality of FP services among others which could not be assessed due to data limitations. Specifically, in-depth assessment of the quality of FP discussions with health care providers could not be conducted in this paper as the NFHS survey does not collect information on the duration of contact and content of the discussions.

## Conclusion

The present study is focused on contraceptive use among married adolescents and the findings offer important policy implications in context of improving adolescent reproductive health. Though there has been an increase in the prevalence of modern contraceptive use, yet only 10% of married adolescents use contraceptives in the country. The same was emphasized during the 2017 Family Planning Summit, which highlighted the slow pace of progression in contraceptive use among adolescents in low and middle-income countries [37]. Majority of adolescents rely either on traditional methods or condoms, both of which have high failure rates. Therefore, interventions to increase awareness among adolescents about the range of available methods and reducing their misconceptions regarding method-related side effects are needed. Local community representatives could be engaged with the adolescents and youth to address their mistrust in contraceptive use. Low rate of contact with health care workers necessitates close monitoring of ongoing FP programs. Further, quality of FP services and counselling should also be assessed regularly as quality of care is an essential predictor for use of FP services. Overall, it is recommended that intervention focused at different levels—health system, community, and adolescents would be a key strategy in delaying child birth and acceptance of contraceptives among adolescents.

## Data Availability

Data would be made available by the corresponding author(s) upon reasonable request.

## Abbreviations

OR: Odds ratio
AOR: Adjusted odds ratio
ASHA: Accredited Social Health Activist
CI: Confidence intervals
FP: Family planning
RMNCH: Reproductive, Maternal, New-born, and Child Health
NFHS: National Family Health Survey
DHS: Demographic and Health Survey
RASTA: Research and Analyses for Scientific Transformation and Advancement
