# Correction to: Contraception for married adolescents (15–19 years) in India: insights from the National Family Health Survey-4 (NFHS-4)

**DOI:** 10.1186/s12978-022-01327-8

**Published:** 2022-02-28

**Authors:** Ijyaa Singh, Ankita Shukla, Jissa Vinoda Thulaseedharan, Gurpreet Singh

**Affiliations:** 1 Achutha Menon Centre for Health Science Studies (AMCHSS), Sree Chitra Tirunal Institute for Medical Sciences and Technology, Trivandrum, Kerala India; 2 Population Council, New Delhi, India

## Correction to: Reproductive Health (2021) 18:253 https://doi.org/10.1186/s12978-021-01310-9

After publication of this article [1], it was reported that in Table 3, heading “Health care worker contacted in the past 3 months”, the confidence interval (1.21, 2.73) should have read (0.86, 1.33).

The original article [1] has been updated.
